# DNA Cleavage, Cytotoxic Activities, and Antimicrobial Studies of Ternary Copper(II) Complexes of Isoxazole Schiff Base and Heterocyclic Compounds

**DOI:** 10.1155/2014/691260

**Published:** 2014-05-08

**Authors:** Vijay Kumar Chityala, K. Sathish Kumar, Ramesh Macha, Parthasarathy Tigulla, Shivaraj

**Affiliations:** ^1^Department of Chemistry, Osmania University, Hyderabad, Andhra Pradesh 500007, India; ^2^Department of Chemistry, University College of Science, Saifabad, Osmania University, Hyderabad, Andhra Pradesh 500004, India

## Abstract

Novel mixed ligand bivalent copper complexes [**Cu. L. A. ClO**_**4**_] and [**Cu. L. A**] where “**L**” is Schiff bases, namely 2-((3,4-dimethylisoxazol-5-ylimino)methyl)-4-bromophenol (DMIIMBP)/2-((3,4-dimethylisoxazol-5-ylimino)methyl)-4-chlorophenol (DMIIMCP), and “**A**” is heterocyclic compound, such as 1,10-phenanthroline (phen)/2,2^1^-bipyridyl (bipy)/8-hydroxyquinoline (oxine)/5-chloro-8-hydroxyquinoline (5-Cl-oxine), have been synthesized. These complexes have been characterized by IR, UV-Vis, ESR, elemental analysis, magnetic moments, TG, and DTA. On the basis of spectral studies and analytical data, five-coordinated square pyramidal/four-coordinated square planar geometry is assigned to all complexes. The ligands and their ternary complexes with Cu(II) have been screened for antimicrobial activity against bacteria and fungi by paper disc method. The antimicrobial studies of Schiff bases and their metal complexes showed significant activity and further it is observed that the metal complexes showed more activity than corresponding Schiff bases. In vitro antitumor activity of Cu(II) complexes was assayed against human cervical carcinoma (HeLa) cancer cells and it was observed that few complexes exhibit good antitumor activity on HeLa cell lines. The DNA cleavage studies have also been carried out on pBR 322 and it is observed that these Cu(II) complexes are capable of cleaving supercoiled plasmid DNA in the presence of H_2_O_2_ and UV light.

## 1. Introduction

Heterocyclic moieties, found in large number of compounds, play an important role in several biological processes. The biological activity of these compounds is mainly dependent on their molecular structures [1–3]. The azomethine group in Schiff bases (–N=CH–) is a significant feature that makes them important compounds owing to their wide range of biological activities [4–7]. Schiff bases are able to inhibit the growth of several animal tumors which can be altered depending upon the type of substituent present on the aromatic rings. Copper plays an essential role in the human organs and the function of copper in the human body is complex and not fully understood [8–11]. The role of Copper is a biocatalyst in the redox reactions. The Cu(II) complexes have demonstrated a wide variety of coordination geometries around Cu(II) atom with N,N and N,O donor ligands and the structure of complex depending on the number, type, and arrangement of ligands about the copper center. The ligands form stable five- or six-membered rings after complexation with the metal ion [12–15]. 8-Hydroxyquinoline and some of its derivatives are monoprotic N,O donor bidentate chelating agents and exhibited substantial cytotoxic activity against cancer cells. The planar nature of “phen” is its ability to participate as either an intercalating or groove-binding agent with DNA [16–19].

The Cu(II) metal complexes may interact with DNA by covalent or noncovalent bindings. The labile part of these complexes is replaced by a nitrogen base of DNA such as guanine via its N7 donor atom. On other hand, the noncovalent DNA interactions include intercalative, electrostatic, and groove binding of cationic metal complexes along the outside of DNA helix. Intercalation involves the partial insertion of aromatic heterocyclic rings between the DNA base pairs. Copper has high Lewis acidity which eventually facilitates the DNA cleavage.

The interesting biological activities associated with Schiff bases and their mixed ligand complexes promoted us to investigate their DNA interactions. The present paper describes the synthesis, characterization, antimicrobial and antitumor activity, and DNA cleavage activity of novel ternary Cu(II) complexes [Cu(DMIIMBP)(phen)ClO_4_](**1**), [Cu(DMIIMBP)(bipy)ClO_4_](**2**), [Cu(DMIIMBP)(oxine)](**3**), [Cu(DMIIMBP)(5-Cl-oxine)](**4**), [Cu(DMIIMCP)(phen)ClO_4_](**5**), [Cu(DMIIMCP)(bipy)ClO_4_](**6**), [Cu(DMIIMCP)(oxine)](**7**), and [Cu(DMIIMCP)(5-Cl-oxine)](**8**).

## 2. Material and Methods

### 2.1. Physical Measurements

^1^H- NMR and ^13^C- NMR spectra of the ligands were recorded on Bruker 400 MHz NMR instrument and using TMS as internal standard. The EI mass spectra were recorded on a VG micromass 7070-H instrument; ESI mass spectra were recorded on VG AUTOSPEC mass spectrometer. Digital conductivity meter of model DI-909 having a dip-type cell was calibrated with KCl solution. Electronic spectra of metal complexes in DMSO were recorded on Schimadzu UV-VIS 1601 spectrophotometer. Magnetic susceptibilities of the complexes were determined on Gouy balance model 7550 using Hg[Co(NCS)_4_] as standard. The diamagnetic corrections of the complexes were computed using Pascal's constants. TG of complexes was carried on Mettler Toledo Star system in the temperature range of 0–1000°C. Melting points of the ligands and decomposition temperature of complexes were determined on Polmon instrument (model number MP-96). IR spectra of the compounds were recorded using KBr pellets in the range (4000–400 cm^−1^) on Perkin-Elmer Infrared model 337. The percentage composition of C, H, and N of the compounds was determined by using microanalytical techniques on Perkin Elmer 240C (USA) elemental analyzer. The EPR spectra of the copper complexes were recorded on EPR Varian-E-112 at room temperature. The percentage composition of metal ions in solid metal complexes was determined by EDTA titration procedure. All the chemicals used were of analytical reagent grade. Solvents such as water, methanol, acetone, petroleum ether, and chloroform were purified by standard procedures [20].

### 2.2. General Procedure for the Synthesis of Isoxazole Schiff Bases

3,4-Dimethyl-5-aminoisoxazole (1.0 mmol) was dissolved in hot methanol to which 5-bromosalicylaldehyde/5-chlorosalicylaldehyde (1.0 mmol) was added and the mixture was refluxed for 2 hours under nitrogen atmosphere. The dark yellow product formed was filtered and washed with petroleum ether and recrystallized from methanol. Purity of the compounds checked by TLC showed single spot in petroleum ether and ethyl acetate (6 : 4) solvent mixture. Yield was 80–85%.

### 2.3. Synthesis of Ternary Cu(II) Metal Complexes

**Scheme 1:**
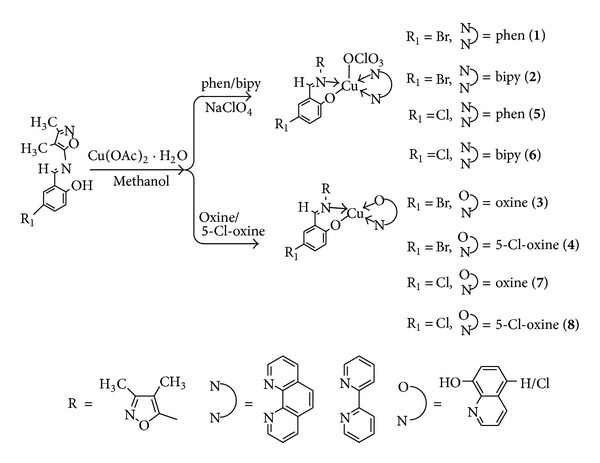
Schematic routes of the synthesis of the complexes.

The synthesis of copper complexes** 1**–**8** was described in Scheme 1. In the preparation of metal complexes, metal to ligands ratio was maintained at 1 : 1 : 1. Hot methanol solution of ligand (1.0 mmol), hot methanol solution of copper acetate monohydrate [Cu(CH_3_COO)_2_·H_2_O] (1.0 mmol), and methanol solution of 1,10-phenanthroline/2,2^1^-bipyridine/8-hydroxyquinoline/5-chloro-8-hydroxyquinoline (1.0 mmol) were mixed together with constant stirring. The mixture was refluxed for 2-3 hours at 70–80°C on water bath. After cooling slowly, a few drops of 0.1 M NaClO_4_ were added for complexes** 1**,** 2**,** 5**, and** 6** with constant stirring over a period of 1 h and on cooling, the dark green/brown coloured metal complexes were precipitated. The products were filtered, washed with cold methanol, and dried under vacuum over P_4_O_10_.

### 2.4. Cell Culture

The human cervical carcinoma cell lines (HeLa) were cultured as a monolayer with Roswell Park Memorial Institute medium (RPMI-1640), supplemented with 10% (v/v) fetal bovine serum (FBS), 2 mM L-glutamine, 4.5 g/L glucose, 1 × nonessential amino acids, and 1 × antibiotics consisting of penicillin/streptomycin, gentamycin, amphotericin B, and nystatin at 37°C, in a humidified atmosphere of 5% CO_2_, in a CO_2_ incubator.

#### 2.4.1. Cytotoxicity Assay (MTT Assay)

The MTT assay was used to assess cytotoxicity [21]. The human cervical carcinoma cell lines (HeLa) were obtained from National Center for Cell Science (NCCS), Pune, India. Briefly, the copper complexes (**1**,** 2**,** 5**, and** 6**) were dissolved in DMSO, diluted in culture medium, and used to treat the cancer cell, with the complex in the concentration range of 2 to 10 *μ*g/mL, for a period of 72 h. DMSO diluted in the culture medium was used as the solvent control. A miniaturized viability assay using 3-[4,5-dimethylthiazol-2-yl]-2,5-diphenyltetrazolium bromide (MTT) was carried out according to the method described earlier [22]. HeLa cells growing exponentially were added to 96-well plates (Orange Scientific) at a density of 3 x 103 per well after counting on Bright Line Haemocytometer (Sigma Ltd.). Compounds (2–10 *μ*g/mL) were then added to the wells, ensuring an equal volume of 200 *μ*L across the plates. Cytotoxicity/proliferation was measured at 72 h using a standard methyl thiazol tetrazolium (MTT) based assay without modifications. Briefly, MTT (Hi Media Ltd.) was added to each well to yield a working concentration of 0.4 mg mL^−1^, and the plates were returned to the incubator for a further 2 h. After this time, the medium was aspirated, 200 *μ*L of DMSO (Sigma Ltd.) was then added to each well, and the plates were agitated gently for 5 min before measuring the optical density at 600 nm in each well using Thermo Scientific Multiskan EX Elisa reader. The IC_50_ value was determined as concentration of the complex that is required to reduce the absorbance to half that of the control.

## 3. Results and Discussion

### 3.1. Characterization of Ligands

The two ligands DMIIMBP and DMIIMCP reported earlier were yellow in colour and stable to air and moisture. The physical and spectral data, such as IR, mass, ^1^H-NMR, and ^13^C-NMR, obtained for these ligands were in good agreement with the literature data [22].

### 3.2. Characterization of Metal Complexes

All the complexes were stable at room temperature and nonhygroscopic. On heating, they were decomposed at high temperatures. The complexes were insoluble in water but were soluble in DMSO. The composition of the complexes was deduced from the elemental analysis. From the data it was clear that the experimental values shown for each of the complexes were in good agreement with the theoretical values calculated for 1 : 1 : 1 ratio (see Section 2.3 of materials and methods). Analytical data of Schiff bases and their metal complexes were presented in Table 1.

### 3.3. IR Spectra

The important infrared spectral bands and their assignments for the synthesized ligands and complexes were recorded as KBr pellets and were presented in Table 2. The IR data of the free ligands and its metal complexes were carried out within the IR range 4000–400 cm^−1^. In all the Schiff bases, azomethine stretching vibrations appear in the 1633–1605 cm^−1^ range. These bands were shifted to lower frequency region to the extent of 10–35 cm^−1^ in complexes, indicating the nitrogen of azomethine is coordinated with the metal ion [23–26]. A broad band around 3453 to 3446 cm^−1^ in ligands due to the phenolic OH group has disappeared in their complexes indicating coordination through phenolic hydroxyl group [27]. A medium intensity band around 1189 to 1159 cm^−1^ due to phenolic *ν*C–O group of the ligands shifted to higher or lower frequency region in their complexes, suggesting the participation of the oxygen of the hydroxyl group in bonding with the metal ion [28, 29]. These facts suggest that these shifts are due to coordination of ligand with the metal atom by the azomethine nitrogen and phenolic oxygen. This fact is also supported by the appearance of nonligand bands at appropriate positions in the far infrared region (528–563 cm^−1^ and 410–454 cm^−1^) due to *ν*M–O and *ν*M–N vibrations, respectively [30–33]. The peaks corresponding to the ring stretching frequencies (*ν*(C=C) and *ν*(C=N)) at 1505, 1421 cm^−1^ of free phen and at 1509, 1423 cm^−1^ of free bipy were shifted to higher frequencies upon complexation (1518, 1430 cm^−1^ for** 1**, 1514, 1446 cm^−1^ for** 2**, 1516, 1431 cm^−1^ for** 5,** and 1516, 1447 cm^−1^ for** 6**), indicating the coordination of the heterocyclic nitrogen atoms of phen and bipy with the metal ion. The characteristic out-of-plane hydrogen bending modes of free phen observed at 851 and 735 cm^−1^ and for bipy at 848, 740 cm^−1^ were shifted to 849, 722 cm^−1^ for** 1**, 841, 731 cm^−1^ for** 2**, 849, 720 cm^−1^ for** 5,** and 830, 30 cm^−1^ for** 6** upon metal complexation [34]. The peaks around 1112, 1104, and 1034 cm^−1^ (*ν*(ClO_4_)) confirm the presence of a coordinated perchlorate ion [35, 36]. A strong *ν*(C–O) band observed in the range between 1105 and 1135 cm^−1^ indicates the presence of oxine moiety in the complexes coordinated through its nitrogen and oxygen atoms as mononegative bidentate ligand [37–40].

### 3.4. Thermal Analysis

**Figure 1:**
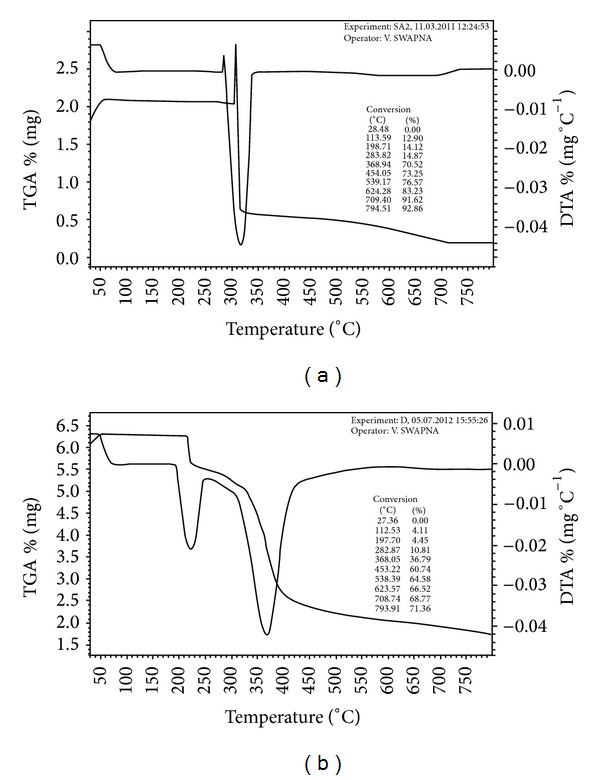
(a) The TGA spectrum of [Cu(DMIIMBP)(phen)ClO_4_]. (b) The TGA spectrum of [Cu(DMIIMCP)(oxine)].

The thermograms of [Cu(DMIIMBP)(phen)ClO_4_] and [Cu(DMIIMCP)(oxine)] were given in Figures 1(a) and 1(b). From the Figures 1(a) and 1(b) it was found that the heating rates were suitably controlled at 10°C min^−1^ under nitrogen atmosphere, and the weight loss was measured from the ambient temperature up to 1000°C. The [Cu(DMIIMBP)(phen)ClO_4_] complex undergoes 1 step of decomposition with the temperature range from 100°C to 370°C with an estimated mass loss of 70.52% (70.91% calculated). In the thermogram it is observed that an exothermic peak appeared at 310°C due to perchlorate. This mass loss corresponds to the pyrolysis of the DMIIMBP and 1,10-phenanthroline ligand molecules leaving CuO as a residue. The [Cu(DMIIMCP)(oxine)] complex undergoes 2 steps of decomposition with the temperature range from 100°C to 370°C with an estimated mass loss of 44.85% (45.01% calculated). This mass loss corresponds to the pyrolysis of the DMIIMCP and oxine ligand molecules leaving CuO as a residue.

### 3.5. Magnetic Susceptibility and Electronic Spectra

The electronic spectral data, molar extension coefficient values, and magnetic susceptibility values of the metal complexes are presented in Table 3 and from the data it is observed that the magnetic moment values of all Cu(II) complexes were in the range of 1.85–1.96 B.M. which is in accordance with one unpaired electron. In the present studies, all Cu(II) complexes show a single broad band in the range from 14,989 to 16,920 cm^−1^ due to transition between ^2^B_1*g*_ → ^2^E_*g*_ suggesting square pyramidal geometry and square planner geometry. Square pyramidal and square planar Cu(II) complexes were expected to give three bands. However, these three bands usually overlap in these complexes to give only one broad absorption band. The electronic spectral and magnetic moment data of all Cu(II) complexes suggest the square pyramidal geometry for complexes** 1**,** 2**,** 5**, and** 6 **and square planar geometry for** 3**,** 4**,** 7**, and** 8** Cu(II) complexes.

### 3.6. ESR Spectra

**Figure 2:**
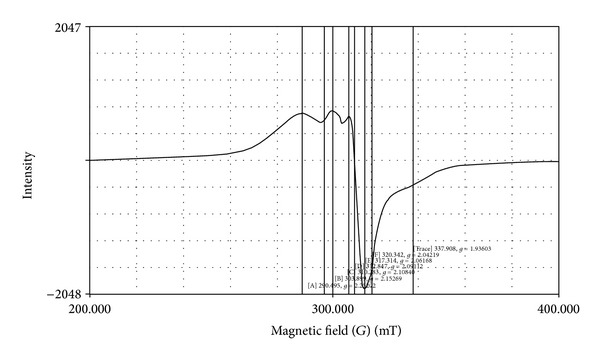
The ESR spectrum of [Cu(DMIIMBP)(phen)ClO_4_].

ESR spectra of all Cu(II) complexes were recorded as polycrystalline sample on X band at frequency 9.3 GHz under the magnetic field strength 3400 G. The ESR data of copper metal complexes** 1**–**8** are presented in Table 4. A representative ESR spectrum of [Cu(DMIIMBP)(phen)ClO_4_] is shown in Figure 2. According to the data *g*_||_ and *g*_⊥_ values for all Cu(II) complexes are found to be in the range of 2.149–2.171 and 2.039–2.061, respectively. From the data, it is clear that *g*_||_ > *g*_⊥_ > 2.0023(*g*_*e*_) which suggest the unpaired electron present in d_*x*2-*y*2_ orbital giving ^2^B_1*g*_ as the ground state and all the complexes are in square planar geometry [41]. Further it was observed that the *g*_||_ values for all Cu(II) complexes were less than 2.3 in agreement with the covalent character of the metal ligand bond. The *g* values are related to axial symmetry parameter *G* by the Hathway expression, that is, *G* = (*g*_||_ − 2.0023)/(*g*_⊥_ − 2.0023).

According to the data, the *G* values for all Cu(II) complexes were found to be less than 4 indicating the ligands were strong field and the metal ligand bonding in these complexes is covalent [42, 43].

## 4. Molecular Modeling

**Figure 3:**
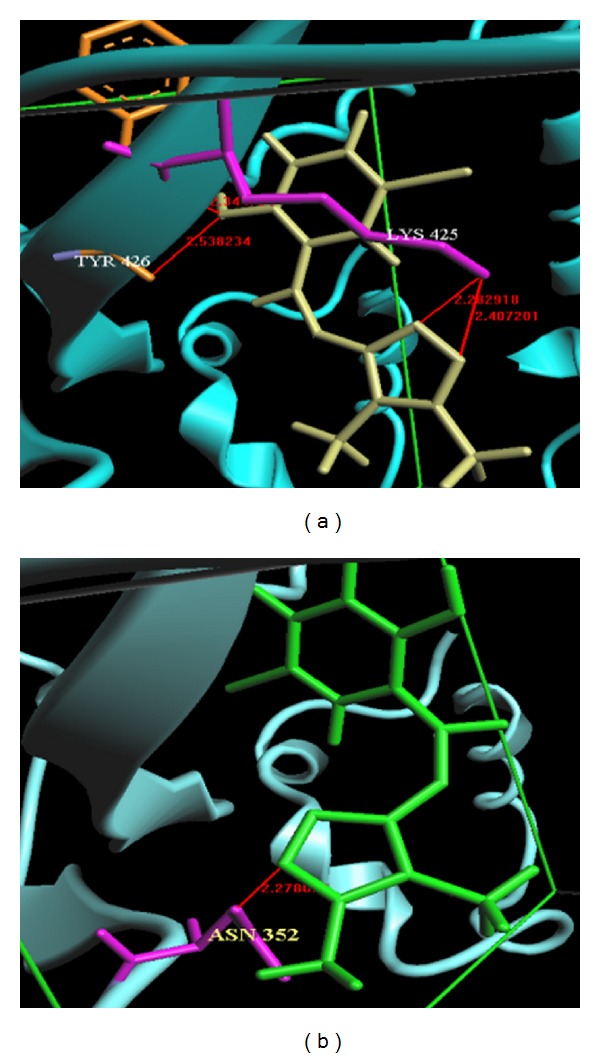
(a) Interactions between TYR and LYS-DMIIMBP crystal ligand (yellow) and target protein (pale green). The TYR residue (gray) and LYS residue (pink) interactions are shown in CPK model. Hydrogen bonding interactions were represented as line (red). (b) Interactions between ASN-DMIIMCP crystal ligand (green) and target protein (pale green). The ASN residue (pink) interactions are shown in CPK model. Hydrogen bonding interactions were represented as line (red).

Successful docking was performed for the selected DMIIMBP and DMIIMCP compounds and their corresponding Gold Fitness score and Chem scores were given in Tables 5(a) and 5(b). The interactions of DMIIMBP and DMIIMCP with protein were shown in Figures 3(a) and 3(b).

**Figure 4:**
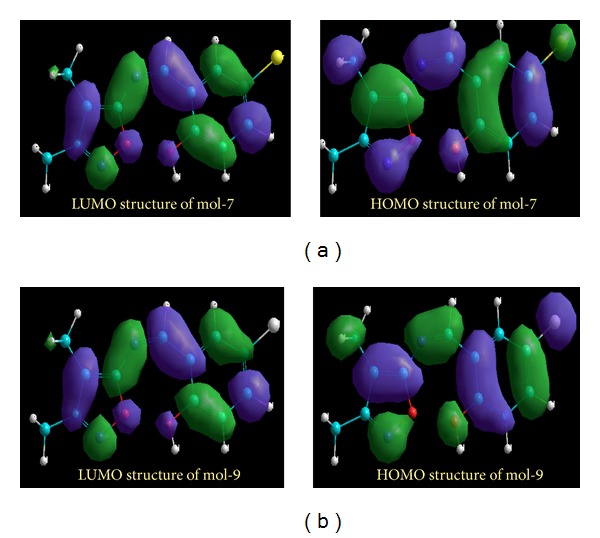
(a) LUMO and HOMO structures for DMIIMBP. (b) LUMO and HOMO structures for DMIIMCP.

For any docked pose [44], the Gold score is calculated generally as the sum of the electrostatic, van der Waals, and hydrophobic interactions and hydrogen bonding interactions. Chem score function includes metal-binding and solvation terms. The Gold Fitness ranges from 57.67 to 57.70 and Chem score ranges from 19.16 to 19.03. Docking results revealed that DMIIMBP and DMIIMCP ligands are involved in hydrogen bonding and van der Waals interactions with the active site residues of DNA topoisomerase I. Among these ligands, DMIIMBP was the best inhibitor of DNA topoisomerase I. The binding energy and the total energy of DMIIMBP and DMIIMCP were listed in Table 6. The LUMO and HOMO structures of DMIIMBP and DMIIMCP were shown in Figures 4(a) and 4(b). The energy levels of LUMO and HOMO reveal that the DMIIMBP was very potential in antimicrobial activity.

## 5. Antimicrobial Activity

In the present investigation, biological activity of the ligands, namely, DMIIMBP, DMIIMCP and their ternary complexes with Cu(II), has been screened for antimicrobial activity against bacteria (*E. coli* and* P. aeruginosa*) and fungi (*A. niger* and* R. oryzae*) by paper disc method. The concentration for these samples was maintained as 1 mg/mL in DMSO. The results thus obtained were explained on the basis of Overtone's concept and Chelation theory [45, 46]. The mode of action of the compounds may involve formation of a hydrogen bond through the azomethine group with the active centers of cell constituents, resulting in an interference with the normal cell process [47].

The variation in the activity of different complexes against different organisms depends either on the impermeability of the cells of the microbes or difference in ribosome of microbial cells. A comparison of the biological activity of the synthesized compounds with some known antibiotics (ciprofloxacin and ketoconazole) is presented in Table 7. It is observed that some of Schiff base metal complexes exhibit better activity than the corresponding ligands.

## 6. Cytotoxic Activity

The cytotoxic activity of Cu(II) complexes was assayed on cultured human cervical carcinoma cell lines (HeLa) for 72 h to the medium containing the respective complexes at 2 to 10 *μ*g/mL concentration and adopting MTT assay and results were given in Table 8.

**Figure 5:**
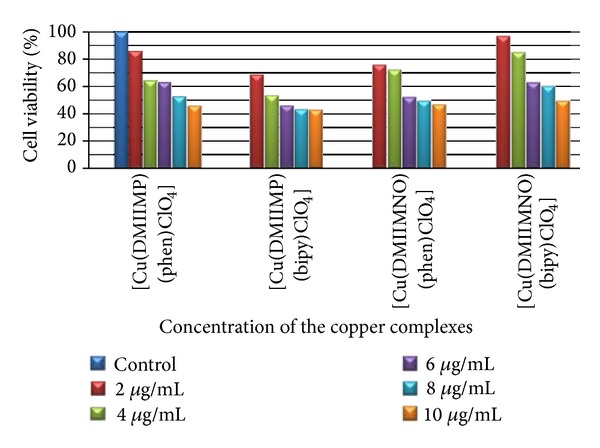
Percentage of cell viability versus different concentrations for HeLa cells exposed to the metal complexes after 72 h incubation.

The cytotoxic activity determined according to the dose values of the exposure of the complex required to reduce survival of the cell was given in Figure 5. The toxicities of 10 *μ*g/mL of [Cu(DMIIMBP)(phen)ClO_4_](**1**), [Cu(DMIIMBP)(bipy)ClO_4_](**2**), [Cu(DMIIMCP)(phen)ClO_4_](**5**), and [Cu(DMIIMCP)(bipy)ClO_4_](**6**) complexes were found to be 54.41%, 57.51%, 52.79%, and 50.22%, respectively. According to these results, [Cu(DMIIMBP)(bipy)ClO_4_](**2**) complex was found to behave as a good antitumor agent on HeLa cell lines. The IC_50_ values obtained in this study were given in Table 9. The IC_50_ value of the [Cu(DMIIMBP)(phen)ClO_4_](**1**), [Cu(DMIIMBP)(bipy)ClO_4_](**2**), [Cu(DMIIMCP)(phen)ClO_4_](**5**), and [Cu(DMIIMCP)(bipy)ClO_4_](**6**) complexes was higher for the 72 h treatment groups, that is, 10 ± 0.09 *μ*g/mL, whereas for [Cu(DMIIMBP)(bipy)ClO_4_] the IC_50_ value is 5 ± 0.06 *μ*g/mL.

## 7. DNA Cleavage Studies

**Figure 6:**
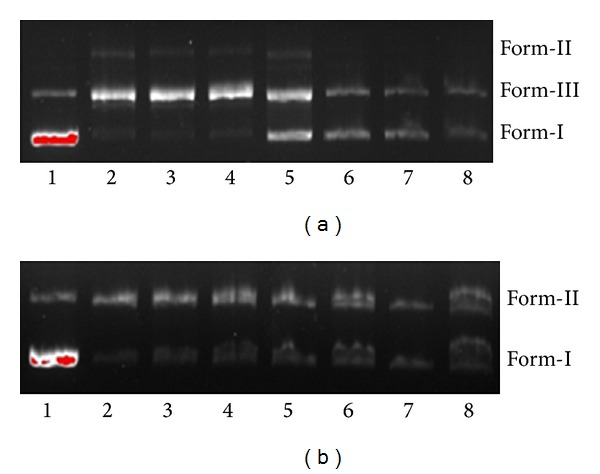
Agarose gel electrophoresis patterns for the oxidative cleavage of pBR 322 DNA by 1 and 2(a) and photolytic cleavage of pBR 322 DNA by 1 and 2(b). (a) Lane 1, DNA control; Lane 2, DNA+**1** (40 *μ*M) + H_2_O_2_ (1 mM); Lane 3, DNA+**1** (80 *μ*M) + H_2_O_2_ (1 mM); Lane 4, DNA+**1** (120 *μ*M) + H_2_O_2_ (1 mM); Lane 5, DNA + H_2_O_2_ (1 mM) + DMSO (1 mM). Lane 6, DNA+**2** (40 *μ*M) + H_2_O_2_ (1 mM); Lane 7, DNA+**2** (80 *μ*M) + H_2_O_2_ (1 mM); Lane 8, DNA+**2** (120 *μ*M) + H_2_O_2_ (1 mM); (b) Lane 1, DNA control; Lane 2, DNA+**1** (40 *μ*M) + H_2_O_2_ (1 mM); Lane 3, DNA+**1** (80 *μ*M) + H_2_O_2_ (1 mM); Lane 4, DNA+**1** (120 *μ*M) + H_2_O_2_ (1 mM); Lane 5, DNA + DMSO (1 mM). Lane 6, DNA+**2** (40 *μ*M) + H_2_O_2_ (1 mM); Lane 7, DNA+**2** (80 *μ*M) + H_2_O_2_ (1 mM); Lane 8, DNA+**2** (120 *μ*M) + H_2_O_2_ (1 mM).

The cleavage activity was demonstrated by gel-electrophoresis experiments using supercoiled (SC) plasmid pBR 322 DNA in a medium TAE buffer. These experiments were monitored by the addition of varying concentrations of the complex (40–200 *μ*M). When DNA was incubated with increasing concentrations of the complexes, SC DNA was degraded to nicked circular (NC) form. The catalytic activities of complexes** 1 **and** 2** were depicted in Figure 6. The activity of** 1** starts at a concentration as low as 40 *μ*M. At 40 *μ*M, 80 *μ*M, and 120 *μ*M, a complete conversion of SC plasmid DNA into the NC form was observed, and the DNA completely smeared is shown in Figure 6(a) (lanes 2, 3, and 4). In contrast, only 63% cleavage was achieved with** 2 **as shown in Figure 6(a) (lanes 6, 7 and 8). This may be due to the efficient binding of** 1** with DNA compared to that of** 2**. To ensure that the Cu complexes were solely responsible for the cleavage, several control experiments were performed under identical conditions. No cleavage of DNA was observed with free Cu (100 mm) and free ligands (100 mm). In a control experiment with DMSO (1 mm), a known radical scavenger, only slight inhibition (ca. 2%) of DNA cleavage was observed, ruling out the possibility of DNA cleavage via OH-based depurination pathway and also a possible oxidative cleavage [48, 49]. When circular plasmid DNA is subjected to electrophoresis, relatively fast migration will be observed for the intact supercoil form (Form-I). If scission occurs on one strand (nicking), the supercoil will relax to generate a slower-moving open circular form (Form-II). If both strands were cleaved, a linear form (Form-III) that migrates between Form-I and Form-II will be generated [50]. The cleavage effect upon irradiation of the plasmid pBR322 DNA in the presence of different concentrations of complexes** 1 **and** 2 **has been tested and is shown in Figure 6(b): Lane 1 DNA alone, Lanes 2–4 DNA + complex (**1**), and Lanes 6–8 DNA + complex (**2**) with increasing concentration (40 *μ*M, 80 *μ*M, and 120 *μ*M) of complex. Form-II increases and Form-I decreases gradually as the concentration of complex increases. The results suggest concentration-dependent single-strand cleavage of supercoiled Form-I to the nicked Form-II. Under comparable experimental conditions, complex** 1** exhibits more effective DNA cleavage activity than complex** 2**. The different cleaving efficiency may be ascribed to the different binding affinity of complexes to DNA.

## 8. Conclusions

Mixed ligand metal chelates of Cu(II) with DMIIMBP/DMIIMCP and phen/bipy/oxine/5-Cl-oxine have been synthesized and characterized. The metal ligand stoichiometry in all complexes was found to be 1 : 1 : 1. The Schiff bases act as monobasic bidentate coordinating through nitrogen of azomethine and phenolic oxygen atom. Based on analytical, conductance, IR, magnetic moments, electronic, and ESR spectral data for the complexes** 1**,** 2**,** 5**, and** 6** and for complexes** 3**,** 4**,** 7**, and** 8**, five-coordinated square pyramidal geometry and four-coordinated square planar geometry are assigned, respectively. Docking studies of the Schiff bases reveal that DMIIMBP Schiff base was more potential for antimicrobial activity. Further it is observed that metal complexes showed higher activity than corresponding Schiff bases and some complexes showed comparable activity with standard drug. The cytotoxicity of all the Cu(II) complexes was assayed and it was found that the cytotoxicity of the [Cu(DMIIMBP)(bipy)ClO_4_] complex at 10 *μ*g/mL concentration shows 57.51% toxicity suggesting good antitumor agent on human cervical carcinoma cell lines. In the DNA cleavage studies it is observed that the [Cu(DMIIMBP)(phen)ClO_4_](**1**) shows more potential activity than the other complexes due to its higher planarity nature.

## Supplementary Material

Contains mole files of ligands **DMIIMBP** and **DMIIMCP**, figure of schematic route of synthesis of complexes, **TG-DTA** spectrum of complex [**Cu(DMIIMBP)(phen)ClO4**] and [**Cu(DMIIMCP)(Oxine)**], the **ESR** spectrum of [**Cu(DMIIMBP)(phen)ClO4**], figures of interactions of **DMIIMBP** and **DMIIMCP** with residues of DNA Topoisomerase I, **HOMO-LUMO** structures of **DMIIMBP** and **DMIIMCP**, figure of cytotoxic activity of complexes on HeLa cells, agarose gel electrophoresis patterns for the oxidative cleavage and photolytic cleavage of pBR 322 DNA by [**Cu(DMIIMBP)(phen)ClO4**](**1**) and [**Cu(DMIIMBP)(bpy)ClO4**].

## Figures and Tables

**Table 1:** Analytical data of Schiff bases and their metal complexes.

Compound	Formula	M.Wt.	C	H	N	O	M
DMIIMBP	C_12_H_11_BrN_2_O_2_	296	48.76 (48.84)	3.58 (3.76)	9.34 (9.49)	10.54 (10.84)	—
[Cu(DMIIMBP)(phen)ClO_4_]	C_24_H_20_BrClCuN_4_O_6_	637	44.69 (45.09)	3.11 (3.15)	8.46 (8.76)	14.81 (15.01)	9.89 (9.94)
[Cu(DMIIMBP)(bipy)ClO_4_]	C_22_H_20_BrClCuN_4_O_6_	613	42.33 (42.94)	3.17 (3.28)	8.94 (9.11)	15.14 (15.60)	10.18 (10.33)
[Cu(DMIIMBP)(oxine)]	C_21_H_18_BrCuN_3_O_3_	502	49.25 (50.06)	3.34 (3.60)	8.27 (8.34)	9.24 (9.53)	12.53 (12.61)
[Cu(DMIIMBP)(5-Cl-oxine)]	C_21_H_17_BrClCuN_3_O_3_	536	46.01 (46.86)	3.09 (3.18)	7.92 (7.81)	8.65 (8.92)	11.02 (11.81)
DMIIMCP	C_12_H_11_ClN_2_O_2_	250	57.05 (57.49)	4.34 (4.42)	11.07 (11.17)	12.24 (12.76)	—
[Cu(DMIIMCP)(phen)ClO_4_]	C_24_H_20_Cl_2_CuN_4_O_6_	593	55.10 (55.46)	3.21 (3.39)	8.99 (9.42)	15.26 (16.14)	10.13 (10.68)
[Cu(DMIIMCP)(bipy)ClO_4_]	C_22_H_20_Cl_2_CuN_4_O_6_	569	41.98 (46.29)	3.05 (3.53)	9.14 (9.81)	15.95 (16.82)	10.82 (11.13)
[Cu(DMIIMCP)(oxine)]	C_21_H_18_ClCuN_3_O_3_	458	54.89 (54.90)	3.85 (3.95)	9.10 (9.15)	10.26 (10.45)	12.99 (13.83)
[Cu(DMIIMCP)(5-Cl-oxine)]	C_21_H_17_Cl_2_CuN_3_O_3_	492	50.99 (51.08)	3.19 (3.47)	8.14 (8.51)	9.11 (9.72)	12.67 (12.87)

**Table 2:** Some important IR absorption frequencies (cm^−1^) of Schiff bases and metal complexes.

Compound	*v*(OH)	*v*(HC=N)	*v*(C–O)	*v*(M–O)	*v*(M–N)	Other bands
DMIIMBP	3446	1641	1182	—	—	*v*(C–Br) 779
[Cu(DMIIMBP)(phen)ClO_4_]	—	1620	1164	550	448	*v*(Cl–O) 1070, 1104, 1148
[Cu(DMIIMBP)(bipy)ClO_4_]	—	1614	1162	548	445	*v*(Cl–O) 1034, 1058, 1110
[Cu(DMIIMBP)(oxine)]	—	1607	1167 and 1113	550	410	—
[Cu(DMIIMBP)(5-Cl-oxine)]	—	1618	1159 and 1133	543	454	*v*(C–Cl) 780
DMIIMCP	3453	1605	1183	—	—	*v*(C–Cl) 778
[Cu(DMIIMCP)(phen)ClO_4_]	—	1622	1164	552	434	*v*(Cl–O) 1071, 1104, 1148
[Cu(DMIIMCP)(bipy)ClO_4_]	—	1618	1162	550	419	*v*(Cl–O) 1036, 1059, 1112
[Cu(DMIIMCP)(oxine)]	—	1609	1189 and 1109	521	414	—
[Cu(DMIIMCP)(5-Cl-oxine)]	—	1633	1183 and 1128	549	421	*v*(C–Cl) 776

**Table 3:** Electronic spectral data and magnetic susceptibility values of the Cu(II) complexes.

Complex	Wave number (*v*) cm^−1^ (nm)	*ε* = 10^2^ M^−1^ cm^−1^	*μ*_eff_ (BM)
[Cu(DMIIMBP)(phen)ClO_4_]	15,545 (646)	0.044	1.85
[Cu(DMIIMBP)(bipy)ClO_4_]	16,323 (615)	0.059	1.95
[Cu(DMIIMBP)(oxine)]	16,891 (592)	0.061	1.92
[Cu(DMIIMBP)(5-Cl-oxine)]	15,954 (627)	0.072	1.89
[Cu(DMIIMCP)(phen)ClO_4_]	15,210 (662)	0.056	1.93
[Cu(DMIIMCP)(bipy)ClO_4_]	14,989 (665)	0.063	1.89
[Cu(DMIIMCP)(oxine)]	16,273 (614)	0.049	1.96
[Cu(DMIIMCP)(5-Cl-oxine)]	16,920 (591)	0.045	1.87

**Table 4:** ESR data of copper metal complexes **1–8**.

Compound	Temperature	*g*_ll_	*g*_⊥_	Δ*g*	*G*
[Cu(DMIIMBP)(phen)ClO_4_]	300 K	2.1539	2.0402	0.1137	3.8279
[Cu(DMIIMBP)(bipy)ClO_4_]	300 K	2.1523	2.0536	0.0987	2.9238
[Cu(DMIIMBP)(oxine)]	300 K	2.1510	2.0391	0.1119	3.8624
[Cu(DMIIMBP)(5-Cl-oxine)]	300 K	2.1497	2.0411	0.1086	3.6437
[Cu(DMIIMCP)(phen)ClO_4_]	300 K	2.1594	2.0431	0.1162	3.6928
[Cu(DMIIMCP)(bipy)ClO_4_]	300 K	2.1710	2.0616	0.1093	2.841
[Cu(DMIIMCP)(oxine)]	300 K	2.1498	2.0423	0.1075	3.5416
[Cu(DMIIMCP)(5-Cl-oxine)]	300 K	2.1526	2.0426	0.1099	3.5807

**Table:** (a)

S. number	Compound	Gold Fitness	S(hb_ext)	S(vdw_ext)	S(hb_int)	S(vdw_int)
1	DMIIMBP	57.67	4.67	41.97	0.00	−4.71
2	DMIIMCP	57.70	4.55	41.52	0.00	−3.94

**Table:** (b)

S. number	Compound	Chem score	DG	S(hbond)	S(metal)	S(lipo)	DE(clash)	DE(int)
1	DMIIMBP	19.16	−19.44	1.88	0.00	87.41	0.03	0.25
2	DMIIMCP	19.03	−19.23	1.90	0.00	85.10	0.01	0.18

**Table 6:** The energy values of the ligands DMIIMBP and DMIIMCP.

Molecule	HOMO (eV)	LUMO (eV)	Binding energy (kcal/mol)	Total energy (kcal/mol)
DMIIMBP	−8.96	−1.09	−7.36	−2959.70
DMIIMCP	−8.89	−1.06	−7.49	−2976.18

**Table 7:** Antimicrobial activity of the Schiff bases and metal complexes.

Complex	*E. Coli *	*P. aeruginosa *	*R. oryzae *	*A. niger *
DMIIMBP	17	14	11	8
[Cu(DMIIMBP)(phen)ClO_4_]	19	16	13	10
[Cu(DMIIMBP)(bipy)ClO_4_]	18	15	12	9
[Cu(DMIIMBP)(oxine)]	16	14	10	8
[Cu(DMIIMBP)(5-Cl-oxine)]	18	17	9	9
DMIIMCP	16	13	10	10
[Cu(DMIIMCP)(phen)ClO_4_]	17	16	11	12
[Cu(DMIIMCP)(bipy)ClO_4_]	18	15	11	11
[Cu(DMIIMCP)(oxine)]	15	14	8	8
[Cu(DMIIMCP)(5-Cl-oxine)]	16	15	9	10
Ciprofloxacin	20	18	—	—
Ketoconazole	—	—	15	13

**Table 8:** In vitro cytotoxicity (HeLa cell line) of Cu (II) complexes.

Concentration (*μ*g/mL)	Absorbance	Cell viability (%)	Toxicities (%)	Concentration (*μ*g/mL)	Absorbance	Cell viability (%)	Toxicities (%)
Control	0.1165	100	0	Control	0.1165	100	0
[Cu(DMIIMBP) (phen)ClO_4_]				[Cu(DMIIMBP) (bipy)ClO_4_]			
2	0.1011	86.90	13.10	2	0.0800	68.66	31.34
4	0.0751	64.35	35.65	4	0.0625	53.64	46.36
6	0.0732	63.05	36.95	6	0.0534	45.83	54.17
8	0.0611	52.50	47.50	8	0.0502	43.09	56.91
10	0.0532	45.59	54.41	10	0.0495	42.49	57.51
[Cu(DMIIMCP) (phen)ClO_4_]				[Cu(DMIIMCP) (bipy)ClO_4_]			
2	0.0880	75.53	24.47	2	0.1120	96.13	3.87
4	0.0833	71.50	28.50	4	0.0985	84.54	15.46
6	0.0605	51.93	48.07	6	0.0726	62.31	37.69
8	0.0580	49.78	50.22	8	0.0696	59.74	40.26
10	0.0550	47.21	52.79	10	0.0580	49.78	50.22

**Table 9:** IC_50_ range of Cu (II) complexes for HeLa cells.

Complex	IC_50_ (*μ*g/mL)
[Cu(DMIIMBP)(phen)ClO_4_]	9 ± 0.03
[Cu(DMIIMBP)(bipy)ClO_4_]	5 ± 0.06
[Cu(DMIIMCP)(phen)ClO_4_]	8 ± 0.03
[Cu(DMIIMCP)(bipy)ClO_4_]	10 ± 0.09
