# Supplementary material for: DNA Cleavage, Cytotoxic Activities, and Antimicrobial Studies of Ternary Copper(II) Complexes of Isoxazole Schiff Base and Heterocyclic Compounds
Source: Bioinorg Chem Appl. 2014 May 8;2014:691260. doi: 10.1155/2014/691260 (PMC4034397; doi:10.1155/2014/691260)

**Supplementary material for review only**

Mole file for Ligand DMIIMBP

Mole file for Ligand DMIIMCP

**Figures in Tiff format**

**Scheme 1.**

**
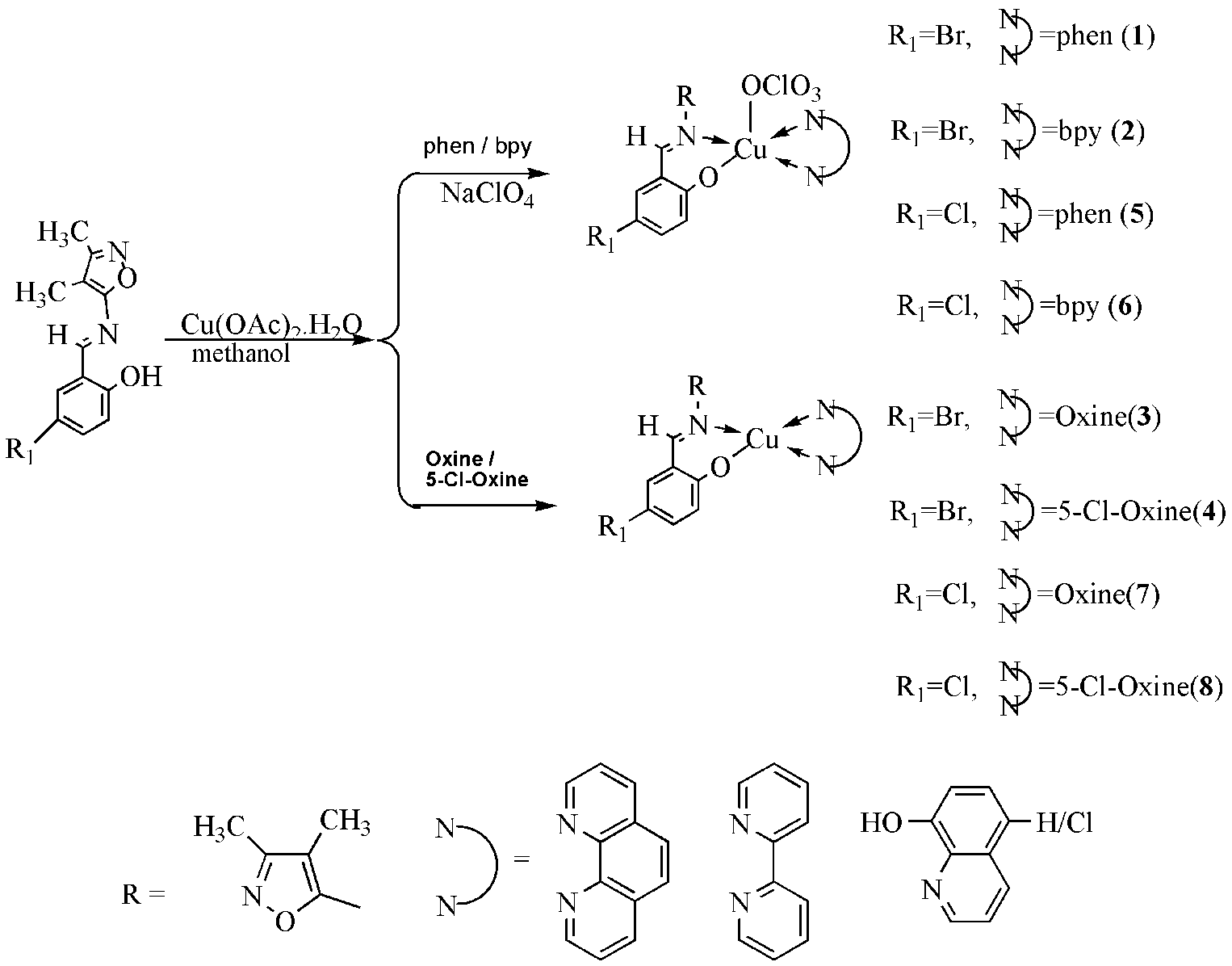
**

**Fig. 1(a).**


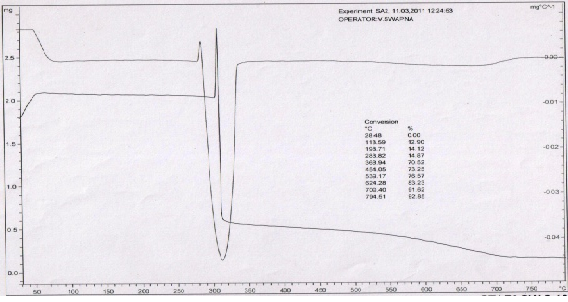


**Fig. 1(b).**


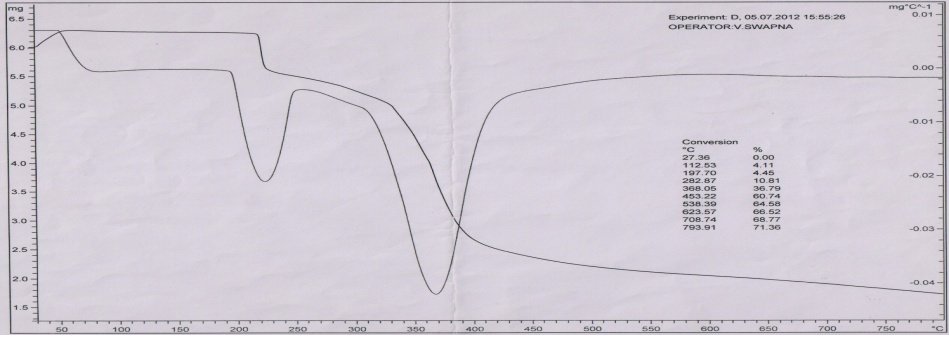


**Fig. 2.**


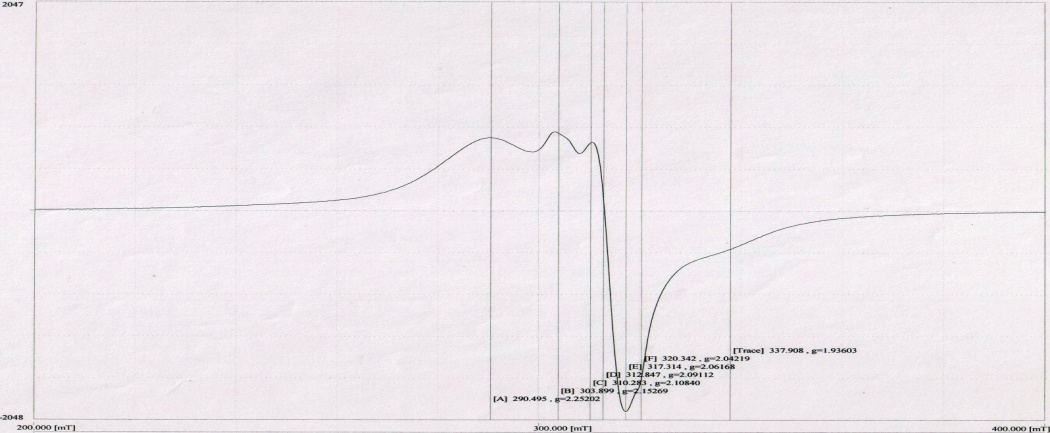


**Figure-3(a)**

**
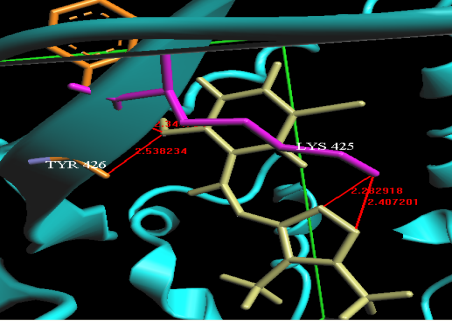
**

**Figure-3(b)**

**
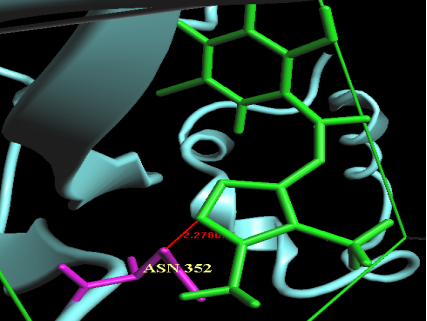
**

**Fig. 4(a).**

**
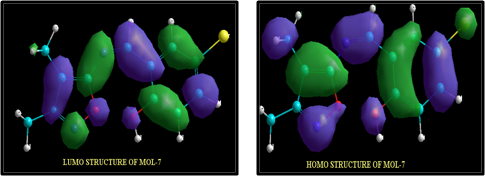
**

**Fig. 4(b).**


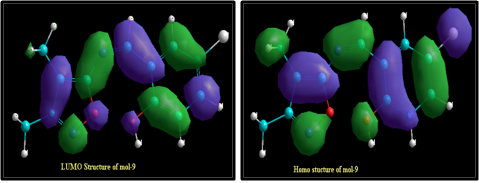


**Fig. 5.**

**
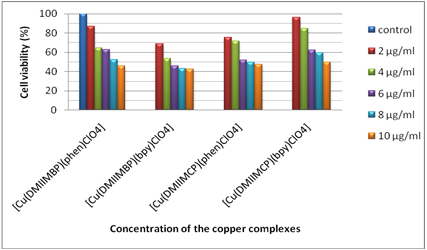
**

**Fig. 6.**


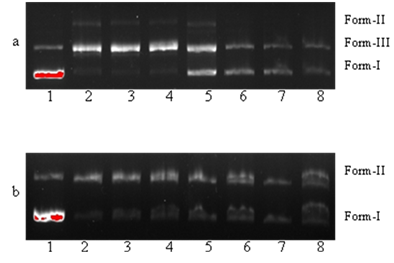

Supplement: Supplementary file 1 — Contains mole files of ligands DMIIMBP and DMIIMCP, figure of schematic route of synthesis of complexes, TG-DTA spectrum of complex [Cu(DMIIMBP)(phen)ClO4] and [Cu(DMIIMCP)(Oxine)], the ESR spectrum of [Cu(DMIIMBP)(phen)ClO4], figures of interactions of DMIIMBP and DMIIMCP with residues of DNA Topoisomerase I, HOMO-LUMO structures of DMIIMBP and DMIIMCP, figure of cytotoxic activity of complexes on HeLa cells, agarose gel electrophoresis patterns for the oxidative cleavage and photolytic cleavage of pBR 322 DNA by [Cu(DMIIMBP)(phen)ClO4](1) and [Cu(DMIIMBP)(bpy)ClO4]. [file 691260.f1.docx]
